# A new computed tomography‐based approach to quantify swallowing muscle volume by measuring tongue muscle area in a single slice

**DOI:** 10.1002/jcsm.13537

**Published:** 2024-07-12

**Authors:** Javier Hurtado‐Oliva, Aniek T. Zwart, Jeroen Vister, Anouk van der Hoorn, Roel J.H.M. Steenbakkers, Inge Wegner, Gyorgy B. Halmos

**Affiliations:** ^1^ Department of Otorhinolaryngology, Head and Neck Surgery University Medical Center Groningen, University of Groningen Hanzeplein 1, PO Box 30.001 9700 RB Groningen The Netherlands; ^2^ Departamento de Fonoaudiología, Facultad de Medicina Universidad de Chile Santiago Chile; ^3^ Department of Radiology University Medical Center Groningen, University of Groningen Groningen The Netherlands; ^4^ Department of Radiation Oncology University Medical Center Groningen, University of Groningen Groningen The Netherlands

**Keywords:** CT scan, dysphagia, head and neck cancer, sarcopenia, swallowing muscle

## Abstract

**Background:**

Measuring the swallowing muscle mass with volume measurements is complex and time intensive; therefore, it is not used in clinical practice. However, it can be clinically relevant, for instance, in the case of sarcopenic dysphagia. The aim of the study was to develop a feasible and clinically applicable method to measure swallowing muscle mass.

**Methods:**

Data from 10 head and neck cancer patients were collected from the Oncological Life Study data‐biobank of the University Medical Center Groningen. The pharyngeal constrictor, genioglossus, mylohyoid and geniohyoid complex muscles, as well as the tongue complex muscles, were delineated manually on routinely performed head and neck computed tomography scans. Axial and sagittal planes were used for volume and area measurements, respectively. Muscle density measurements were performed with and without Hounsfield unit thresholding. Correlations were assessed by Pearson correlation coefficients, and interobserver reliability was measured using intra‐class correlation coefficients (ICCs).

**Results:**

Significant differences were observed between sagittal area measurements with and without Hounsfield unit thresholds for pharyngeal constrictor, tongue complex and the sum of the swallowing muscles (*t* > 6; *P*‐value < 0.001). Stronger correlations emerged without Hounsfield unit thresholding. Strong positive and significant correlations were found between the total swallowing muscle mass volume and the sagittal area of the tongue complex muscles (*r* = 0.87, *P*‐value < 0.05) and the sum of the sagittal areas of the pharyngeal constrictor and tongue complex muscles (*r* = 0.85, *P*‐value < 0.05). The use of the Hounsfield unit threshold weakened correlations. Interobserver reliability was assessed and found to be fair to good for the pharyngeal constrictor muscle (ICC = 0.68, *P*‐value < 0.05), excellent for the tongue complex muscles (ICC = 0.98, *P*‐value < 0.05) and excellent for the total swallowing muscle area (ICC = 0.96, *P*‐value < 0.05).

**Conclusions:**

Single‐slice delineation of the sagittal area of tongue complex muscle and pharyngeal constrictor muscle is a promising, fast, simple and clinically applicable method for measuring the total volume of the swallowing muscle mass in head and neck cancer patients without Hounsfield unit thresholding. These advancements and findings would help in the early and accurate diagnosis of definitive sarcopenic dysphagia.

## Introduction

Swallowing is a complex process, and several muscles are involved in the swallowing action.^1^, ^2^ The evaluation of the swallowing process involves essential aspects of muscles, including their mass and strength, as both parameters play a crucial role in facilitating effective swallowing.^3^ Decreased swallowing muscle mass (SwMM) is known to be associated with several conditions, like neurological diseases,^4^ but also as a treatment‐related complication, for instance, after therapy for head and neck cancer (HNC).^5^ Moreover, a decrease in SwMM is also known to be related to aging and sarcopenia.^6^, ^7^

Swallowing disorders and sarcopenia are common in HNC patients and also in the general elderly population. As a consequence, these conditions often coexist in these populations because of weight loss, loss of skeletal muscle mass (SMM) and decline in physical and physiological functions.^8^ Early and accurate diagnosis of sarcopenic dysphagia would be essential for early rehabilitation and the prevention of health‐related adverse consequences.

There are several methods known to measure muscle mass using ultrasound,^9^ computed tomography (CT)^5^, ^10^, ^11^ and magnetic resonance imaging (MRI).^12^, ^13^ However, none of these measurements are routinely performed in clinical practice due to their complexity, as all these methods require both expertise and time, potentially facing limitations in accessibility and implementation across various healthcare services.^6^, ^14^ Furthermore, there is no consensus on cut‐off values, and there is a lack of studies that verify the reliability and validity of diagnostic tools that measure SwMM volume.^15^, ^16^, ^17^

Imaging of the head and neck with CT is often part of the routine oncological workup of HNC. Therefore, SwMM, as measured with conventional neck CT, may be a practical tool to identify HNC patients at risk for sarcopenic dysphagia that can easily be integrated into daily clinical practice.

The aim of the present feasibility study was to develop a simple, less time‐intensive and therefore more feasible and reliable method to measure the SwMM in HNC, fitting into the diagnostics in clinical practice by (1) measuring the total volume of SwMM in HNC, (2) investigating the correlation between the total volume of the SwMM and the sagittal area of the SwMM and (3) assessing the interobserver reliability of the sagittal area of the SwMM measurements.

## Materials and methods

### Ethical considerations

Data from the present study were retrospectively collected from the Oncological Life Study (OncoLifeS), a large prospective oncological data‐biobank approved by the Medical Ethical Committee of the University Medical Center Groningen (UMCG).^18^ OncoLifeS prospectively includes patients over the age of 18 who have been diagnosed with cancer. Patients are included after informed consent. OncoLifeS aims to (1) provide an infrastructure for clinical cancer research, (2) use translational research to set the stage for personalized patient care and (3) monitor and improve the quality of care. This and more information can be found on the website of OncoLifeS (https://umcgresearch.org/w/oncolifes). OncoLifeS is registered in the Trial Register of the Netherlands under Registration Number NL7839. The study protocol was approved by the scientific board of OncoLifeS. All data and used CT scans were anonymized.

### Study population

We included 10 patients from the OncoLifeS data‐biobank, diagnosed with a primary or recurrent mucosal or advanced cutaneous malignancy in the head and neck area at the Department of Otorhinolaryngology/Head and Neck Surgery and the Department of Oral and Maxillofacial Surgery of the UMCG between October 2014 and April 2016. All of them were randomly reselected from previous published studies for inclusion.^19^, ^20^, ^21^, ^22^ A sample size of 10 patients was chosen because we wanted to explore the feasibility of measuring SwMM and because of the time‐consuming nature of the volume measurements that had to be performed by an experienced head and radiologist. All patients underwent a pre‐treatment neck CT scan as part of the normal oncological workup. Patients whose scans were very oblique or had poor image quality due to motion or streak artefacts were excluded. Cases in which swallowing muscles were affected by the tumour, such as tumour muscle infiltration, were also excluded. Baseline patients' characteristics retrieved from the data‐biobank were age (years), sex, weight (kg), length (m), body mass index (BMI; kg/m^2^), localization of the tumour and stage of the disease. Tumour staging was performed according to the TNM Classification of Malignant Tumours from the Union for International Cancer Control, 8th edition.^23^

### Measurements of the swallowing muscles

Muscle analysis was performed using the Aquarius workstation Intuition Edition (ver.4.4.13.P6) program on post‐contrast neck CT scans reconstructed with a 1.0‐mm slice thickness and a soft‐tissue kernel. Two independent observers participated in the SwMM measurements: a board‐certified head and neck radiologist (JV), and a speech and language therapist/PhD student (JH‐O). The latter was trained using a training dataset, and the observer's performance was tested before performing the measurements in this study cohort to ensure clarity and reproducibility of the method. Observer JV performed the volume measurements, and both observers (JV and JH‐O) performed the area measurements of the same set of CT scans blinded to each other's outcome to calculate interobserver reliability (see further details on interobserver reliability under the Statistical analysis section). The mean area measurements of the two observers were used for the correlation analyses with the swallowing muscle volume. To correctly measure the volume and area of the swallowing muscles by the observers, a step‐by‐step manual was generated in this present study (see supporting information for details).

**Figure 1:**
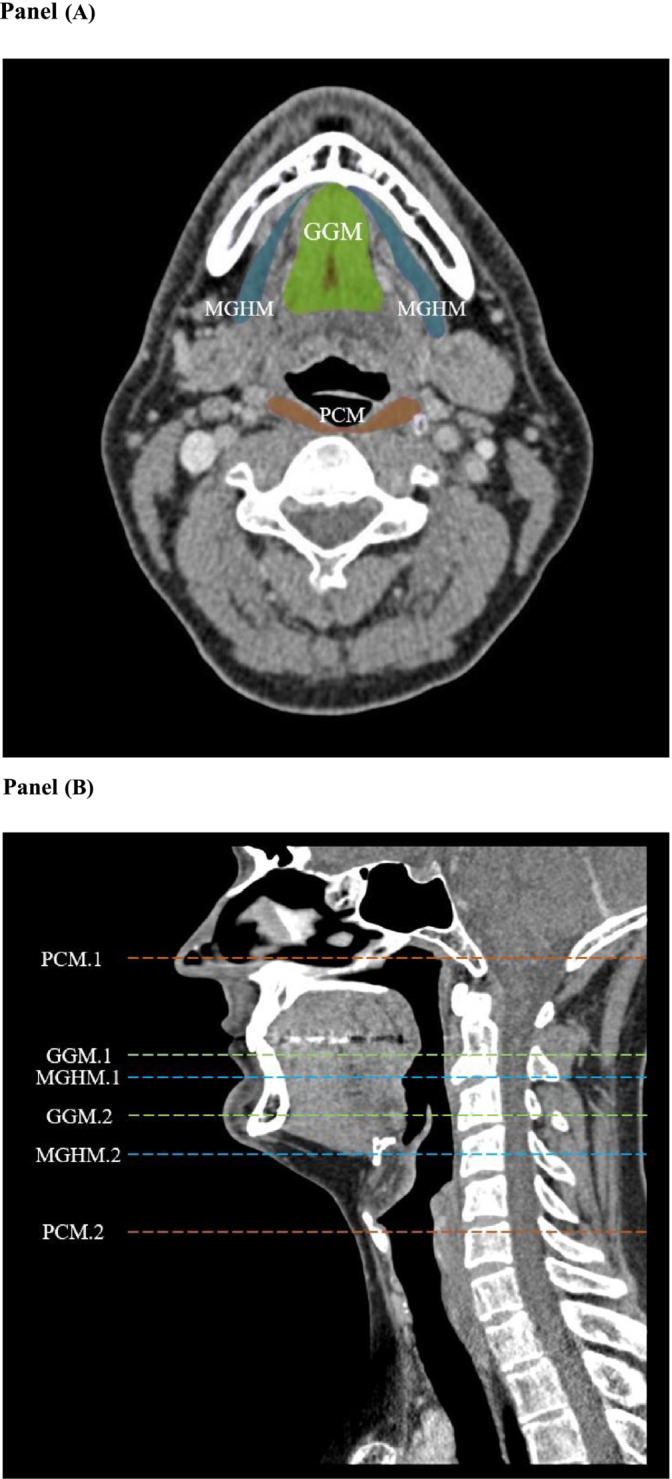
Example of the swallowing muscle mass volume delineations and measurements. (A) Axial plane at level of the third cervical vertebra (C3). The orange, green and blue colour areas represent the delineated muscles. (B) Sagittal plane, superior and inferior muscle borders according to anatomical landmarks. GGM, genioglossus muscles; GGM.1, superior border genioglossus muscles; GGM.2, inferior border genioglossus muscles; MGHM, mylohyoid and geniohyoid muscle complex; MGHM.1, superior border mylohyoid and geniohyoid muscle complex; MGHM.2, inferior border mylohyoid and geniohyoid muscle complex; PCM, pharyngeal constrictor muscles; PCM.1, superior border pharyngeal constrictor muscles; PCM.2, inferior border pharyngeal constrictor muscles.

Volume measurements of the muscles involved a semi‐automatic delineation of the region of interest on axial slices. For muscles, this involves identifying muscle borders and separating them from surrounding tissues. Once the segmentation is complete, the software compiles the outlined regions from multiple slices to create a 3D volume rendering of the muscle mass. The interpolated delineation provides a comprehensive measure of the entire structure. Based on the study of Dale et al., the following dysphagia‐related muscles were selected for analysis: the pharyngeal constrictor muscles (PCM), genioglossus muscles (GGM) and mylohyoid and geniohyoid muscle complex (MGHM).^10^ The selection of these muscles was driven by their importance in swallowing and their susceptibility to loss of muscle mass, as indicated by previous studies.^12^, ^13^, ^24^, ^25^ The total SwMM volume was defined as the summed volume of the PCM, GGM and MGHM. The delineation process involved identifying key anatomical landmarks for each muscle group to ensure precision. For delineating the PCM, we utilized the clivus, vocal cords (with use of the cuneiform tubercles), pharyngeal airway and cervical spine. Each landmark served as a reference for the superior, inferior, anterior and posterior borders, respectively. The MGHM delineation utilized the mandible, hyoid and GGM as anatomical borders. The measurement of the GGM was done by eyeballing the anterior, posterior and lateral muscle borders. For an illustrative overview, see *Figure* *1*, which provides a visual representation of the muscle delineation.

**Figure 2:**
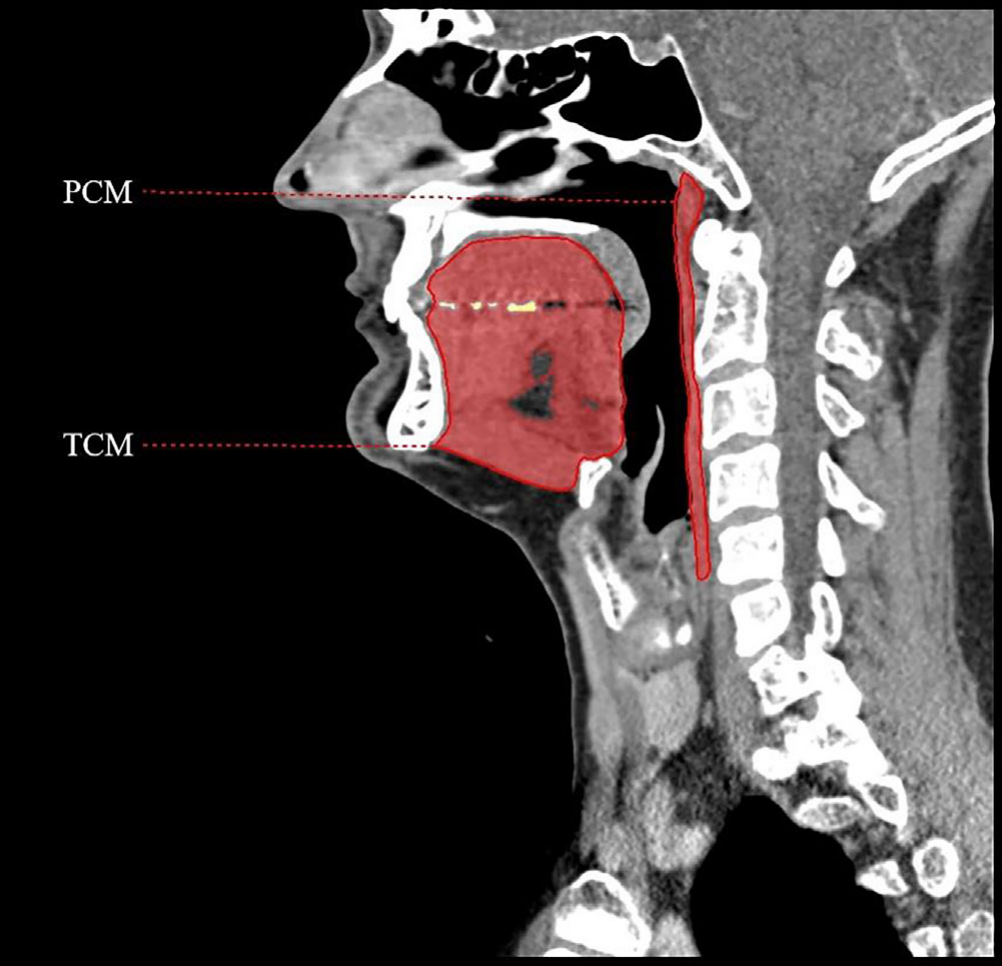
Example of the swallowing muscle mass area measurements on a sagittal computed tomography slice. Anterior, posterior, superior and inferior muscle borders identified according to anatomical landmarks. The red area represents the delineated muscles. PCM, pharyngeal constrictor muscles; TCM, tongue complex muscles.

Before initiating the area measurements in the sagittal plane, slice selection using a fixed angulation of the anatomical planes had to be performed. First, the X‐axis of the sagittal plane was rotated and fixed perpendicular to the posterior border of the pharyngeal airway. Afterwards, the Y‐axis of the axial and coronal planes was rotated and aligned perpendicular to the base of the pterygoid bone and to the bony hard palate. Then, the most medial sagittal slice was selected to perform the area measurements. The previously mentioned anatomical landmarks of the muscles were identified prior to the delineation of the area of the PCM, GGM and MGHM. However, it was difficult to reliably reproduce the upper border of the GGM and differentiate the GGM from the surrounding muscles. Therefore, we decided to delineate and measure the whole tongue complex muscles (TCM), including the GGM, MGHM and intrinsic tongue musculature, to facilitate identification of its borders and to make it easier for practical use. The anatomical landmarks for the TCM were the muscle insertion at the mandible, the tongue base, the inferior border of the MGHM and the oral cavity for the anterior, posterior, inferior and superior borders, respectively. The vallecula, epiglottis and oral vestibule were excluded from the TCM. The total SwMM area was defined as the summed area of the PCM and TCM. See *Figure* *2* for the muscle area measurements.

**Figure 3:**
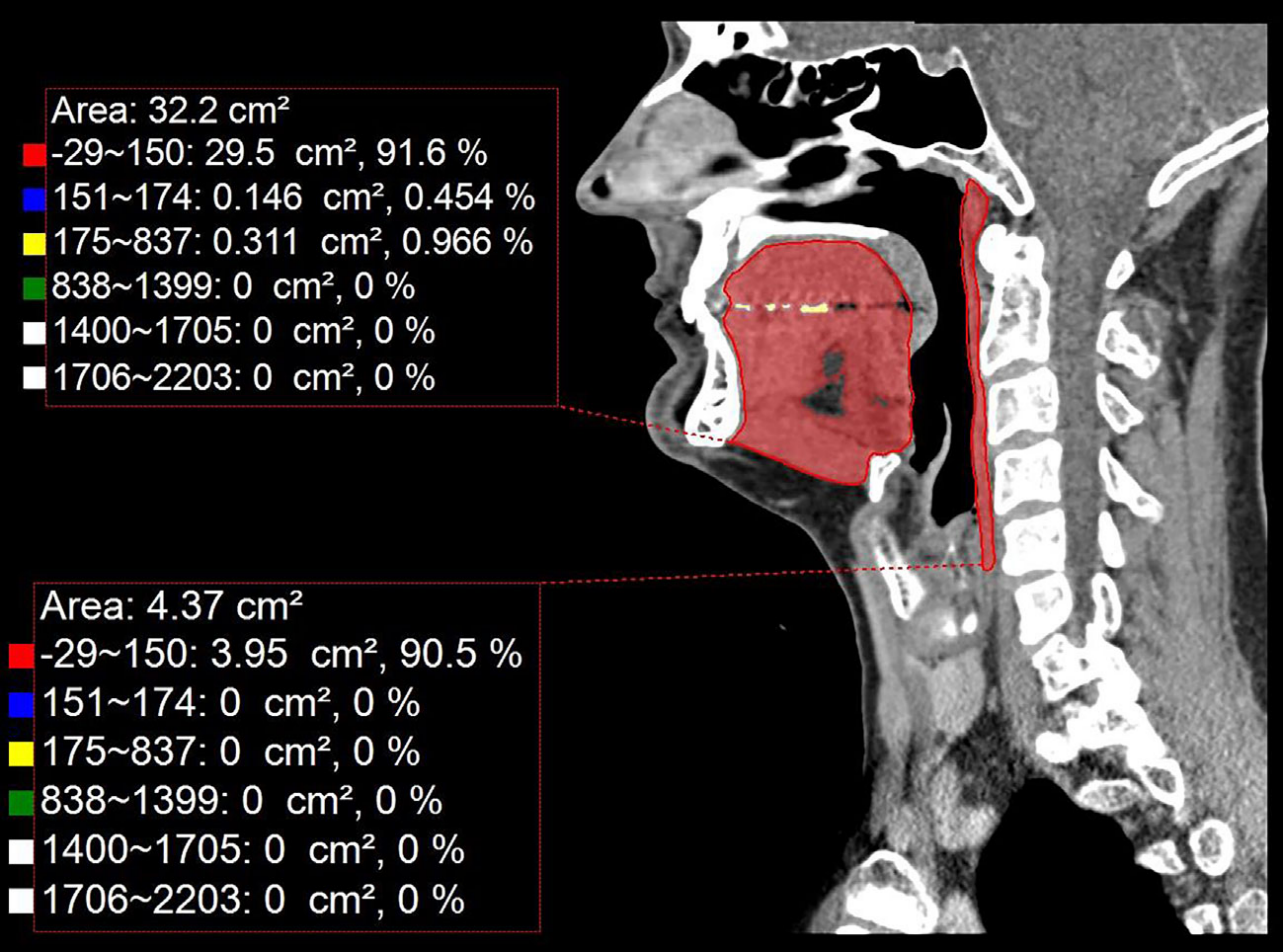
Example of the swallowing muscle mass area measurements with and without applying Hounsfield unit (HU) thresholding. The upper measurement (area: 32.2 cm^2^) corresponds to the sagittal area of the tongue complex muscle without the HU threshold. The lower measurement (area: 4.37 cm^2^) corresponds to the sagittal area of the pharyngeal constrictor muscle without the HU threshold. The value marked with a red square (−29 ~ 150) corresponds to the measured muscle area with HU thresholding. Other HU ranges correspond to different muscle densities.

After muscle delineation, the outcomes were presented with and without Hounsfield unit (HU) thresholding for both the volume and area measurements. The HU tool would help to isolate and quantify the region of interest, allowing us to focus on specific tissues with particular density characteristics. For HU thresholding, the range was set from −29 to +150 HU, which corresponds with muscle density.^26^ This set‐up and measurement would allow for a more specific examination of muscle characteristics based on their density and provide information about the identification of specific muscle‐related conditions or abnormalities (*Figure* *3*).

### Statistical analyses

Statistical analyses were performed using Statistical Package for the Social Sciences (SPSS) statistics software Version 28.0 (IBM Corp., Armonk, NY, USA). Categorical variables were presented as frequencies with corresponding percentages, and continuous variables as mean scores with their standard deviations (SDs).

Normality distribution of volume and area muscle measurements was assessed using the Shapiro–Wilk test and visual inspections of Q–Q plots. Therefore, a paired‐samples *t*‐test was conducted to compare the volume of the swallowing muscles and the sagittal area of the swallowing muscles with and without using an HU threshold for muscle density. The effect size was analysed using Cohen's *d* and Hedge's correction. Correlations between the swallowing muscle volume and sagittal area of the swallowing muscles were assessed using Pearson correlation coefficients. Interobserver reliability was measured for PCM, TCM and the total SwMM area using intra‐class correlation coefficients (ICCs) on all scans using a two‐way mixed single measures model with absolute agreement. The ICCs were rated as poor (0.00–0.49), fair to good (0.50–0.74), good (0.75–0.90) and excellent (>0.90).^27^

## Results

### Patient characteristics

The mean age of the population was 65.9 (±13.2) years, and the majority was male (70%). Most of the patients had oropharyngeal cancer (40%), and seven patients (70%) had stage III disease. Regarding the histopathology, nine patients (90%) had a squamous cell carcinoma, and one patient (10%) had an angiosarcoma. Patient and tumour characteristics are presented in *Table* *1*.

**Table 1:** Demographic and clinical characteristics

Variable	*N* (%)
Age (mean, years, SD)	65.9 (±13.2)
Sex	
Male	7 (70%)
Female	3 (30%)
BMI (kg/m^2^, SD)	26.3 (±4.8)
Tumour localization	
Lip and oral cavity	2 (20%)
Oropharynx	4 (40%)
Larynx	2 (20%)
Skin	2 (20%)
Stage	
I	2 (20%)
II	1 (10%)
III	7 (70%)
IV	0
Histopathology	
Squamous cell carcinoma	9 (90%)
Other	1 (1%)

Abbreviations: BMI, body mass index; SD, standard deviation.

### Radiological findings

#### Volume measurements of the swallowing muscles

The mean volumes of the SwMM with and without HU thresholding were 65.12 cm^3^ (±16.01) and 71.20 cm^3^ (±17.09), respectively. There was a strong and statistically significant difference between these measurements (*t* > 4; *P*‐value < 0.001). Moreover, looking at the volume measurements of the PCM, GGM and MGHM separately, similar results were found. The effect size analysed using Cohen's *d* and Hedge's correction was >2.0 for PCM and for the SwMM, suggesting a large difference between the means of the compared groups. The results are presented in *Table* *2*.

**Table 2:** Volume and sagittal area measurements of the swallowing muscles

Measurement	Muscles	With HU Th	Without HU Th	Paired‐samples *t*‐test				Effect size	
		Mean (SD)	Mean (SD)	Mean difference (SD)	*t*‐value	df	*P*‐value	Cohen's *d*	Hedge's correction
Volume (cm^3^)	PCM	24.62 (5.63)	28.30 (6.76)	3.68	4.892	9	<0.001	2.3	2.4
	GGM	21.12 (8.17)	22.07 (8.40)	0.95	6.496	9	<0.001	0.4	0.4
	MGHM	19.38 (5.93)	20.83 (6.20)	1.44	6.643	9	<0.001	0.6	0.7
	All muscles	65.12 (16.01)	71.20 (17.09)	6.07	6.632	9	<0.001	2.8	3.0
Area (cm^2^)	PCM	1.86 (0.63)	3.85 (1.06)	1.98	6.286	9	<0.001	0.9	1.0
	TCM	27.26 (5.43)	31.98 (5.91)	4.71	8.449	9	<0.001	1.7	1.8
	All muscles	29.13 (5.69)	35.83 (6.57)	6.69	11.7	9	<0.001	1.8	1.8

Abbreviations: GGM, genioglossus muscles; HU Th, Hounsfield unit threshold; MGHM, mylohyoid and geniohyoid muscle complex; PCM, pharyngeal constrictor muscles; SD, standard deviation; TCM, tongue complex muscles.

#### Sagittal area measurements of the swallowing muscles

The mean area of the SwMM with and without HU thresholding was 29.13 cm^2^ (±5.69) and 35.83 cm^2^ (±6.57), respectively. There was also a strong and statistically significant difference between the measurements while using an HU threshold and the measurements without using an HU threshold when measuring the PCM, TCM and sum of the swallowing muscles (*t* > 6; *P*‐value < 0.001). There was a large difference between the means of the compared groups, according to Cohen's *d* and Hedge's correction. The results are presented in *Table* *2*.

#### Correlation between the sagittal area and volume of the swallowing muscles

The correlations between the sagittal area and volume measurements were generally stronger when the HU threshold was not used compared with when the HU threshold was used (*Table* *3*).

**Table 3:** Pearson correlation coefficient between swallowing muscle volume and sagittal area

Swallowing muscle volume	Sagittal area of swallowing muscles (*R*; CI)					
	PCM		TCM		Total SwMM area	
	Without HU Th	With HU Th	Without HU Th	With HU Th	Without HU Th	With HU Th
PCM						
Without HU Th	0.80 (0.335 to 0.950)	0.61 (−0.037 to 0.895)	0.71 (0.140 to 0.925)	0.80 (0.345 to 0.951)	0.77 (0.261 to 0.941)	0.83 (0.422 to 0.959)
With HU Th	0.80 (0.336 to 0.950)	0.57 (−0.100 to 0.881)	0.69 (0.112 to 0.921)	0.82 (0.380 to 0.955)	0.75 (0.233 to 0.938)	0.84 (0.446 to 0.961)
GGM						
Without HU Th	0.12 (−0.551 to 0.697)	0.31 (−0.396 to 0.787)	0.76 (0.243 to 0.939)	0.60 (−0.043 to 0.893)	0.70 (0.125 to 0.923)	0.61 (−0.032 to 0.896)
With HU Th	0.11 (−0.560 to 0.691)	0.32 (−0.385 to 0.792)	0.74 (0.205 to 0.934)	0.58 (−0.075 to 0.887)	0.68 (0.092 to 0.918)	0.59 (−0.061 to 0.890)
MGHM						
Without HU Th	0.11 (−0.556 to 0.693)	0.16 (−0.521 to 0.718)	0.60 (−0.057 to 0.891)	0.36 (−0.351 to 0.806)	0.55 (−0.119 to 0.877)	0.36 (−0.350 to 0.806)
With HU Th	0.15 (−0.531 to 0.712)	0.21 (−0.483 to 0.742)	0.63 (0.008 to 0.903)	0.41 (−0.295 to 0.827)	0.60 (−0.056 to 0.891)	0.42 (−0.291 to 0.828)
Total SwMM volume						
Without HU Th	0.42 (−0.290 to 0.828)	0.45 (−0.249 to 0.842)	0.87 (0.524 to 0.968)	0.74 (0.213 to 0.935)	0.85 (0.466 to 0.963)	0.76 (0.248 to 0.940)
With HU Th	0.39 (−0.317 to 0.819)	0.44 (−0.260 to 0.838)	0.86 (0.491 to 0.965)	0.74 (0.198 to 0.933)	0.83 (0.427 to 0.959)	0.75 (0.230 to 0.937)

Abbreviations: CI, confidence interval; GGM, genioglossus muscles; HU Th, Hounsfield unit threshold; MGHM, mylohyoid and geniohyoid muscle complex; PCM, pharyngeal constrictor muscles; SwMM, swallowing muscle mass; TCM, tongue complex muscles.

**Figure 4:**
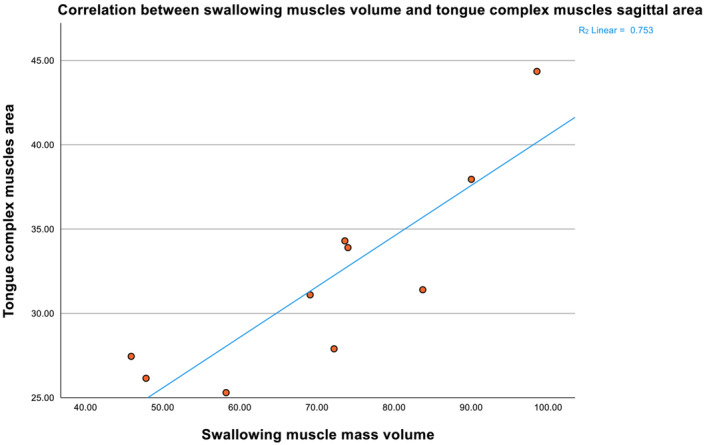
Scatter plot correlation between swallowing muscle mass volume and tongue complex muscle mass area.

The correlations between the sagittal area measurements and PCM volume measurements without using an HU threshold were consistently strong, positive and statistically significant (*r* = 0.71 to 0.80, *P*‐value < 0.05). The total SwMM area correlated strongly with the GGM volume measurements and the total SwMM volume (*r* = 0.70 and *r* = 0.85, *P*‐value < 0.05, respectively). The TCM sagittal area measurements also correlated strongly with the GGM volume measurements and the total SwMM volume (*r* = 0.76 and *r* = 0.87, *P*‐value < 0.05, respectively). A visual representation of the correlation trends between TCM sagittal area measurement and the total SwMM volume is provided in *Figure* *4*.

When using the HU threshold for sagittal area and volume measurements, most of the correlations were weaker. The correlations between the sagittal area measurements of the TCM and the volume measurements of the PCM and the total SwMM volume were rather strong (*r* = 0.82 and *r* = 0.74, *P*‐value < 0.05, respectively), as well as the correlation between the total SwMM area and the volume measurements of the PCM and the total SwMM volume (*r* = 0.84 and *r* = 0.75, *P*‐value < 0.05, respectively).

Regardless of using the HU threshold, there were weak to moderate correlations between all sagittal area measurements and the MGHM volume measurements (*r* = 0.11 to 0.63). Correlations between the PCM sagittal area measurement and all volume measurements were weak (*r* = 0.11 to 0.61) except for the correlation between the PCM sagittal area measurements without using an HU threshold and PCM volume measurements (*r* = 0.80, *P*‐value < 0.05).

#### Interobserver reliability

The ICC for the area measurements of the PCM was 0.68 (*P*‐value < 0.05), indicating fair to good agreement between observers. The area measurements of the TCM showed an ICC of 0.98 (*P*‐value < 0.05), indicating excellent agreement between observers. Additionally, the ICC for the total SwMM area was 0.96 (*P*‐value < 0.05), also indicating excellent agreement between observers (*Table* *4*).

**Table 4:** Interobserver reliability of the swallowing muscle area

Muscles	Intra‐class correlation (*R*)	95% CI		*P*‐value
		Lower bound	Upper bound	
PCM	0.681	−0.242	0.929	<0.05
TCM	0.980	0.880	0.996	<0.001
Total SwMM area	0.959	0.292	0.992	<0.001

Abbreviations: CI, confidence interval; PCM, pharyngeal constrictor muscles; SwMM, swallowing muscle mass; TCM, tongue complex muscles.

## Discussion

This is the first study demonstrating a simple and reliable method to measure SwMM. Our findings revealed a strong, positive and statistically significant correlation between the TCM sagittal area measurement and the total SwMM volume (*r* = 0.87), as well as between the total SwMM area and the total SwMM volume (*r* = 0.85), without using the HU threshold. The interobserver reliability was excellent for the TCM sagittal area measurement with an ICC of 0.98, as well as for the total SwMM area measurement with an ICC of 0.96. These results show that TCM sagittal area measurement seems to be a feasible proxy for the total SwMM volume and affirm its simplicity, which is crucial for facilitating widespread adoption in routine clinical practice in favour of diagnostic accuracy.

The relevance of imaging assessment in determining muscle mass for dysphagia‐related studies is highlighted by several recent studies. Ultrasound imaging was utilized in studies by Ogawa et al.^9^ and Mori et al.^28^ to examine SwMM and its association with sarcopenic dysphagia, revealing smaller tongue muscle mass and emphasizing the predictive value of geniohyoid muscle mass for tongue pressure and overall muscle mass. Umay et al.^29^ further emphasized the importance of imaging by identifying a decrease in masseter thickness as an independent risk factor for dysphagia in older adults. Ultrasound has predominantly focused on examining the geniohyoid, tongue complex and digastric muscles in healthy young and elderly adults, aiming to establish reliability data, compare age‐related changes and investigate the relationship between tongue thickness and nutritional status.^24^, ^30^, ^31^

On the other hand, CT scans were employed by Dale et al.,^10^ Hashida et al.^11^ and Garber et al.^5^ to assess radiation dose–response thresholds, predict dysphagia severity after radiation therapy and salvage surgery and evaluate tongue volume changes after chemo‐radiotherapy, respectively. Additionally, CT scans were also used to explore the association between radiation dose to PCM and swallowing dysfunction and the cross‐sectional area of geniohyoid muscle atrophy with aging and aspiration.^25^, ^32^ Nakao et al.^13^ utilized MRI to understand age‐related composition changes in swallowing‐related muscles, highlighting the vulnerability of the geniohyoid muscle to muscle atrophy. Moreover, Molfenter et al.^12^ underscored the importance of assessing pharyngeal muscle thickness and lumen size due to age‐related changes.

These previous studies emphasize the importance of assessing SwMM. Although ultrasound has been proposed as a convenient, cost‐effective and radiation‐free method with the possibility to measure muscle morphology and quality,^28^ its complex methodology and need for additional assessment procedure details in the current research studies hinder its practical application in clinical settings. Notably, this method lacks the capability to assess PCM, crucial for swallowing performance, with evidence that suggests age‐related atrophy of these muscles.^33^, ^34^, ^35^, ^36^ In contrast, our study introduces a straightforward approach and a detailed protocol, ensuring replicability. Furthermore, CT scans are an integral part of the routine oncological workup, providing an opportunity for not only implementing the diagnosis process in daily clinical practice but also opening up the possibility for the development of automated delineation and measurement techniques. Finally, we assessed the mass of the whole group of muscles with a higher propensity for atrophy due to age and sarcopenia.

On the other hand, in the study of Dale et al.,^10^ swallowing muscles were delineated in planning CT scans. It is known that these scans are usually low‐dose scans, providing lower resolution and therefore less reliable outcomes. Furthermore, delineation was performed by autosegmentation, reviewed by radiation oncologists. In the study of Hashida et al.,^11^ only the geniohyoid and masseter muscles were delineated, and other swallowing muscles were not included. In contrast, in our study, we included and delineated additional swallowing muscles that may increase the risk of dysphagia as a consequence of their decrease in muscle mass.^6^, ^37^, ^38^ The 3D volume measurement of the tongue muscles on MRI scans by Nakao et al.^13^ methodologically seems to be very secure; however, it is very complicated, time‐consuming and needs to be done by a radiologist, which makes it clinically less applicable for clinicians. On the contrary, our study describes a reliable, reproducible and relatively simple, therefore clinically applicable, method to measure SwMM. Beyond its clinical utility, our study has the potential to expand the dimensions of sarcopenic dysphagia research.

Using an HU threshold when delineating the swallowing muscles led to smaller areas and volumes compared with not using an HU threshold. The delineated total SwMM area was, on average, 7 cm^2^ smaller when using the HU threshold. The delineated total SwMM volume was, on average, 6 cm^3^ smaller when using the HU threshold. It can be explained by technical artefacts that were observed related to the angulation/reconstruction of the sagittal slices and beam‐hardening artefacts. These artefacts led to the exclusion of smaller areas, which appeared as voids within the larger delineated area of muscle mass on CT when using the HU threshold. Therefore, the HU threshold should be carefully considered when performing muscle assessment on CT scans.

The main strength of this study lies in its secure methodology. All CT scans were diagnostic scans with high resolution, 1 mm of slices, and assessed by two independent observers, both well trained, and the interobserver agreement was excellent. Including scans of patients and non‐healthy individuals further strengthens the study, as it better simulates the target population.

However, there are some limitations to consider. First, the study focused only on HNC patients, and the applicability of the proposed method to other populations needs to be investigated. Second, due to the limited sample size, the findings of the study may have limited reliability and generalizability, as the results are more susceptible to variability and may not accurately represent the population. Third, the position of the tongue may affect the TCM measurements; however, there is no literature data on this specific issue. In other muscle mass measurements, the position of body parts did not seem to affect the measurements. For instance, skeletal muscle index (SMI; cm^2^/m^2^) measurements at the level of the third cervical vertebra were found to be independent from the position of the arms.^22^ In addition, the study acknowledged the challenges associated with using HU thresholding and the potential for technical artefacts in the CT scans. Further refinement of the measurement technique may be warranted to overcome these limitations. Lastly, this method can be clinically broader and applied in everyday practice if cut‐off values are established.

The findings of this study open up opportunities for future research in the field of sarcopenic dysphagia and its management in HNC patients. Extending the described method to other imaging modalities, like, for instance, MRI, could provide complementary information and enhance the accuracy of SwMM measurements. As mentioned, it is essential to establish a cut‐off point for SwMM measurements, advancing towards a definitive diagnosis of sarcopenic dysphagia. Finally, the development of automated algorithms for SwMM delineation and analysis could streamline the process, reduce the need for manual measurements and improve efficiency in clinical practice.

## Conclusions

This study presents a simple and feasible method for measuring SwMM using a conventional CT scan. The findings demonstrate a strong correlation between the sagittal area measurement of the TCM and the volume measurement of the sum of swallowing muscles, indicating the potential of TCM sagittal area as a proxy for volume measurements. These advancements hold promise for early and accurate diagnosis of definitive sarcopenic dysphagia, leading to improved management and prevention of health‐related adverse consequences in patients. However, further research is required to validate and refine the proposed method and enhance its clinical applicability.

## Conflict of interest statement

Javier Hurtado‐Oliva declared receiving grant support from the Department of Human Capital Development, Chilean National Scholarship Program for Graduate Studies, of the National Agency of Research and Development (ANID), Government of Chili (Scholarship ID: 72210512). Aniek T. Zwart, Jeroen Vister, Anouk van der Hoorn, Roel J. H. M. Steenbakkers, Inge Wegner and Gyorgy B. Halmos declare that they have no conflict of interest.

## Supporting information


**Data S1.** Supporting Information.
