# Supplementary material for: A new computed tomography‐based approach to quantify swallowing muscle volume by measuring tongue muscle area in a single slice
Source: J Cachexia Sarcopenia Muscle. 2024 Jul 12;15(5):1858–68. doi: 10.1002/jcsm.13537 (PMC11446713; doi:10.1002/jcsm.13537)
Supplement: Supplementary file 1 — Data S1. Supporting Information. [file JCSM-15-1858-s001.pdf]

Cite as: Hurtado-Oliva J., Zwart A. T., Vister J., van der Hoorn A., Steenbakkers R. J. H. M., Wegner I., Halmos G. B. (2024) A new computed tomography-based approach to quantify swallowing muscle volume by measuring tongue muscle area in a single slice, Journal of Cachexia, Sarcopenia and Muscle, doi: https://doi.org/10.1002/jcsm.13537

Supplementary material 1.

Step-by-step manual for CT-scan slice selection and muscles area delineation

I. General information about Aquarius software and data loading

1) Open Aquarius software.
2) Load CT-scan according to patient ID (anonymized). Depending of the anonymized ID coding of included patients, apply filters by patient ID and CT scan modality to find the list of patients included.
3) Double click on the patient's ID. You will see the whole list of imaging exams for the selected patient.
4) At the list of all the scans made For the selected patient, double-click on CT-scan of 1mm thickness.
5) Double click on the CT-scan, and a panel with axial, coronal and sagittal CT-scan planes will open. Then you will be able to navigate through all of them (Figure A).

II. Mid-sagittal CT-scan slice selection

1) In sagittal plane, tilt the Y axis until it is parallel to the posterior border of the airway (figure B).
2) In axial plane, tilt the X-axis according to the base of the pterygoid bone (figure C).
3) In coronal plane, tilt the X-axis according to the bony hard palate (figure D).
4) In axial and coronal plane, place the Y-axis in the middle of head and neck, taking into account the airway, the spine, and the orientation of the head.
5) Double click at the sagittal CT-scan. The best mid-sagittal slice was selected.

III. Muscles area delineation of the pharyngeal constrictor muscle

1) Keep the same angulation in every plane. No changes must be made.
2) Before starting with the delineation of the pharyngeal constrictor muscle (PCM) in sagittal plane, the lower border of the muscle must be identified.
3) In axial plane, identify the trachea. Move upwards from caudal to cranial, until the first scan with both arytenoids is found.
4) In sagittal plane, according to the angulation of X-axis, define the lower border of the PCM at the pharyngeal wall (figure E).
5) In sagittal plane, start with the muscle delineation.

Cite as: Hurtado-Oliva J., Zwart A. T., Vister J., van der Hoorn A., Steenbakkers R. J. H. M., Wegner I., Halmos G. B. (2024) A new computed tomography-based approach to quantify swallowing muscle volume by measuring tongue muscle area in a single slice, Journal of Cachexia, Sarcopenia and Muscle, doi: https://doi.org/10.1002/jcsm.13537

6) Activate polygon (area histogram) in Aquarius software (figure F).
7) With left mouse button can be selected the region of interest with intervals. Double click on left mouse button will end the measurement. It is possible to alter the measurement by clicking on the left mouse button on the white dots.
8) Perform the delineation following the next anatomical landmarks: the clivus, vocal cords (with use of the cuneiform tubercles), pharyngeal airway, and cervical spine for respectively the superior, inferior, anterior, and posterior border (figure G).
9) Once finished, click right mouse button in the red square of measurements and edit color map. Set Hounsfield Unit threshold between -29 until 150 (figure H).
10) Register the measurements: area of the muscle (cm2), and the area according to the Hounsfield Unit threshold (figure I).

IV. Muscles area delineation of the tongue complex muscle

1) Keep the same angulation in every plane. No changes must be made.
2) In sagittal plane, start with the muscle delineation.
3) Activate polygon (area histogram) in Aquarius software (figure F).
4) With left mouse button can be selected the region of interest with intervals. Double click on left mouse button will end the measurement. It is possible to alter the measurement by clicking on the left mouse button on the white dots.
5) Perform the delineation following the next anatomical landmarks: include the genioglossus muscle, mylohyoid and geniohyoid muscles complex, and intrinsic tongue musculature; identify the muscle insertion at the mandible, the tongue base, the inferior border of the MGHM, and oral cavity for respectively the anterior, posterior, inferior, and superior borders. Exclude the vallecula, epiglottis, and oral vestibule from the muscle delineation (figure G).
6) Once finished, click right mouse button in the red square of measurements and edit color map. Set Hounsfield Unit threshold between -29 until 150 (figure H).
7) Register the measurements: area of the muscle (cm2), and the area according to the Hounsfield Unit threshold (figure I).

Cite as: Hurtado-Oliva J., Zwart A. T., Vister J., van der Hoorn A., Steenbakkers R. J. H. M., Wegner I., Halmos G. B. (2024) A new computed tomography-based approach to quantify swallowing muscle volume by measuring tongue muscle area in a single slice, Journal of Cachexia, Sarcopenia and Muscle, doi: https://doi.org/10.1002/jcsm.13537

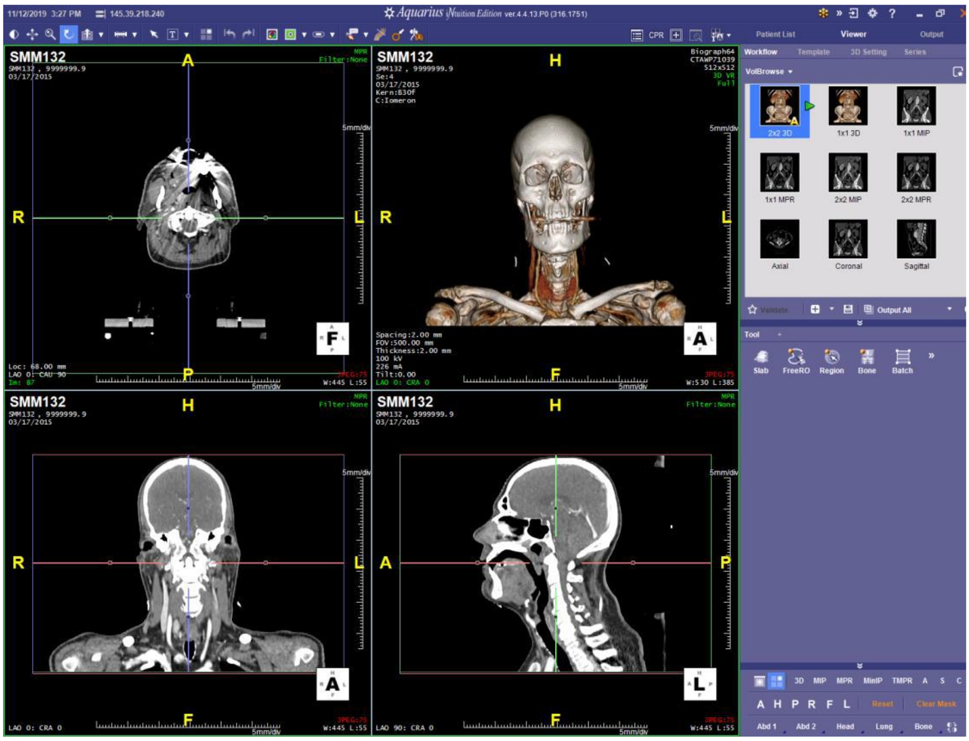

Figure A

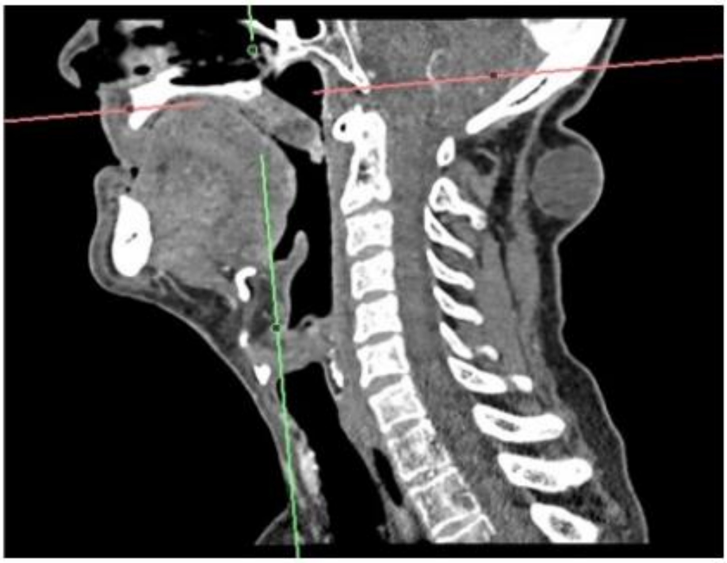

Figure B

Cite as: Hurtado-Oliva J., Zwart A. T., Vister J., van der Hoorn A., Steenbakkers R. J. H. M., Wegner I., Halmos G. B. (2024) A new computed tomography-based approach to quantify swallowing muscle volume by measuring tongue muscle area in a single slice, Journal of Cachexia, Sarcopenia and Muscle, doi: https://doi.org/10.1002/jcsm.13537

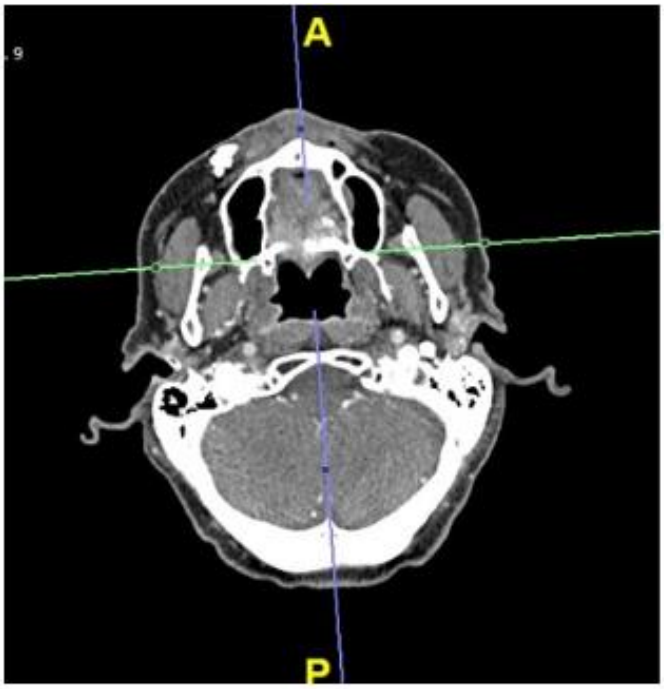

Figure C

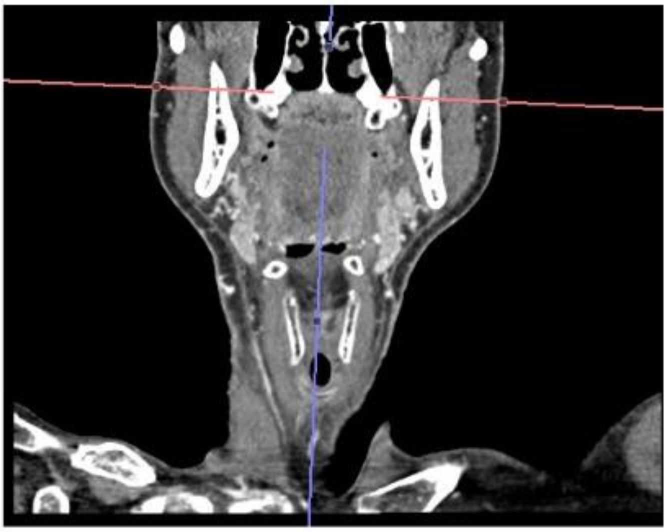

Figure D

Cite as: Hurtado-Oliva J., Zwart A. T., Vister J., van der Hoorn A., Steenbakkers R. J. H. M., Wegner I., Halmos G. B. (2024) A new computed tomography-based approach to quantify swallowing muscle volume by measuring tongue muscle area in a single slice, Journal of Cachexia, Sarcopenia and Muscle, doi: https://doi.org/10.1002/jcsm.13537

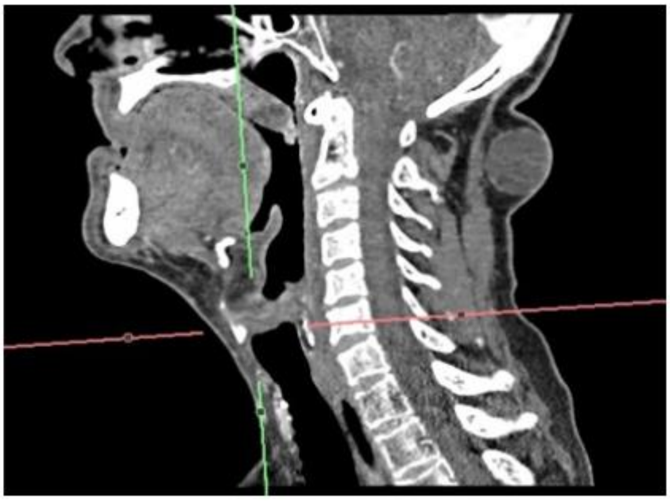

Figure E

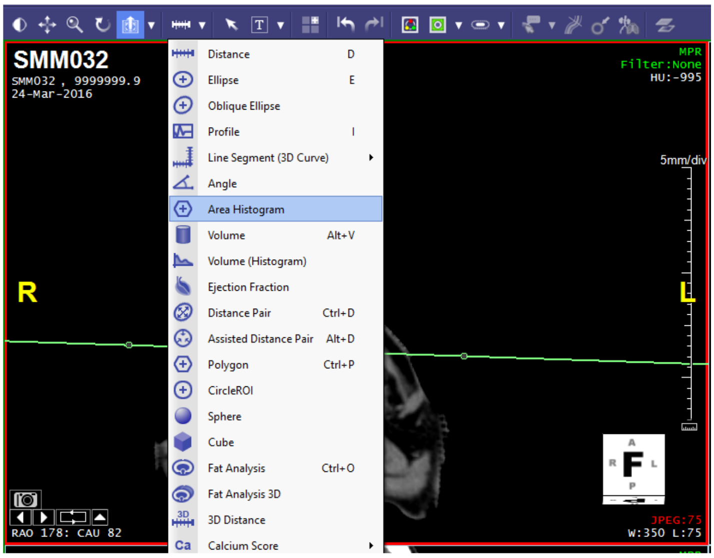

Figure F

Cite as: Hurtado-Oliva J., Zwart A. T., Vister J., van der Hoorn A., Steenbakkers R. J. H. M., Wegner I., Halmos G. B. (2024) A new computed tomography-based approach to quantify swallowing muscle volume by measuring tongue muscle area in a single slice, Journal of Cachexia, Sarcopenia and Muscle, doi: https://doi.org/10.1002/jcsm.13537

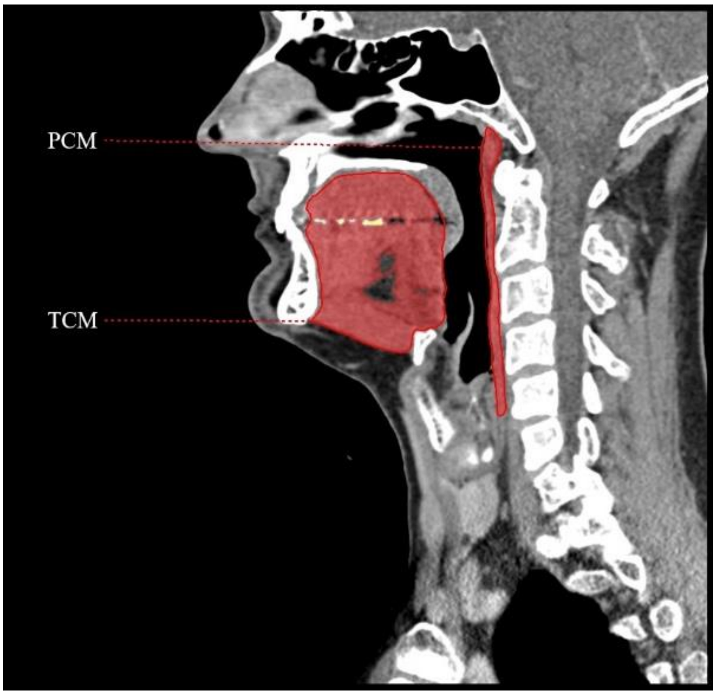

Figure G

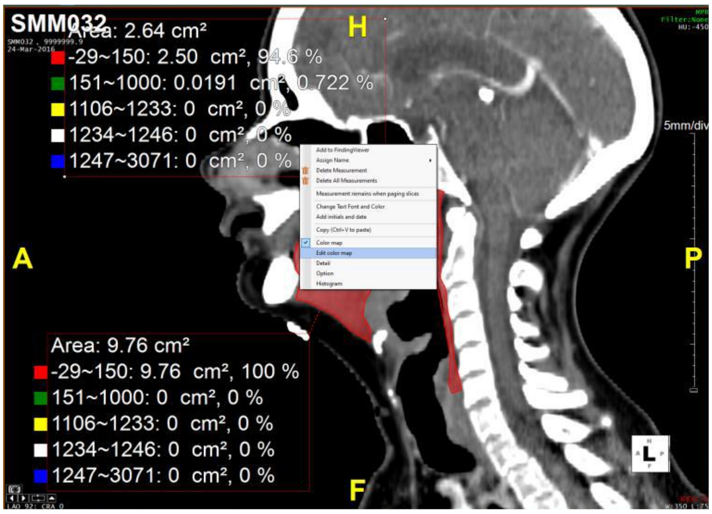

Figure H

Cite as: Hurtado-Oliva J., Zwart A. T., Vister J., van der Hoorn A., Steenbakkers R. J. H. M., Wegner I., Halmos G. B. (2024) A new computed tomography-based approach to quantify swallowing muscle volume by measuring tongue muscle area in a single slice, Journal of Cachexia, Sarcopenia and Muscle, doi: https://doi.org/10.1002/jcsm.13537

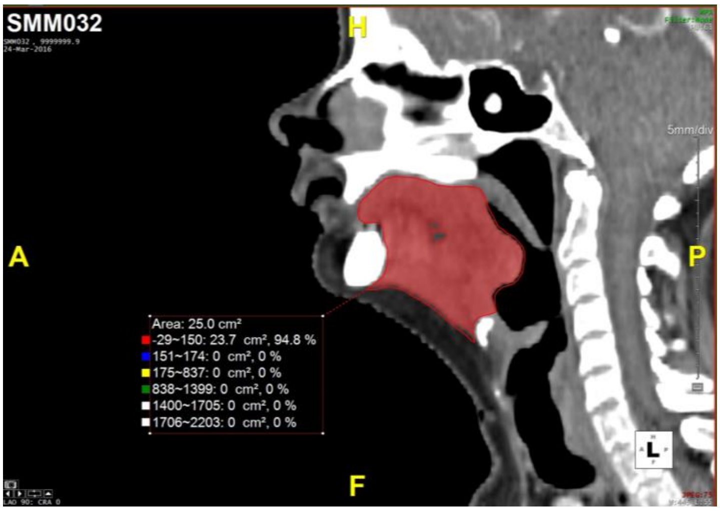

Figure I
